# Combination of bortezomib plus ruxolitinib in steroid-refractory chronic graft-versus-host disease

**DOI:** 10.1038/s41409-018-0362-7

**Published:** 2018-10-18

**Authors:** Panayotis Kaloyannidis, Eshrak Al Shaibani, Ioannis Apostolidis, Solaf Kanfar, Khalid Al Anezi, Hani Al Hashmi

**Affiliations:** Adults Hematology and Stem Cell Transplantation Department, King Fahad Specialist Hospital, Dammam, Saudi Arabia

**Keywords:** Medical research, Haematological diseases

Steroid-refractory graft-versus-host disease (SR-GvHD) is a leading cause for late non-relapse mortality and contributes to high morbidity rates and poor quality of life post allogeneic stem cell transplantation (alloSCT). Although numerous treatment approaches have been proposed and studied in clinical trials [calcineurin inhibitors (CNIs), mycophenolate mofetil (MMF), extracorporeal photopheresis (ECP), anti-thymocyte globulin, mTOR-inhibitors, monoclonal antibodies etc.], SR-GvHD management still remains suboptimal [1, 2]. Recently, new agents such as kinase inhibitors or nuclear factor kappa-B inhibitors have been introduced in the “pharmaceutical arm-depot” for the treatment of SR-GvHD. Roxulitinib, a selective Janus kinase (JAK) 1/2 inhibitor, approved by U.S. Food and Drug Administration (FDA) as second line treatment for the refractory acute-GvHD, has also demonstrated encouraging results in SR-cGvHD [3]. Bortezomib, a proteasome-inhibitor, which currently represents an essential component of multiple myeloma treatment, in phase I–II clinical trials and case reports, has shown noticeable activity either for the prevention or for the treatment of cGvHD [4]. Nevertheless, even in the era of newly approved agents for cGvHD treatment, for a considerable number of patients with SR-cGvHD, the success of second line therapies is limited. Therefore, still there is an unmet need for novel, more effective approaches.

Herein, we present the clinical course and outcome in a patient who developed SR-cGvHD, which demonstrated refractoriness when bortezomib or ruxolitinib were administered as single agents but experienced a meaningful response to the bortezomib plus ruxolitinib combination, suggesting a potential synergistic effect of these two agents. Patient had previously signed an informed consent for the anonymous use of the medical data, including photos, for scientific purposes.

A 22-year-old male was allografted for hepatosplenic gamma-delta T-cell lymphoma, conditioned with the total body irradiation plus cyclophosphamide regimen. The graft source was peripheral blood stem cells from his full-matched sibling sister. The combination of cyclosporine plus short course (four doses) methotrexate was administered as GvHD prophylaxis. The early course post transplant was uneventful; however, 2 months post allograft, the immunosuppression was tapered rigorously because of molecularly detectable residual disease. Two months later, he achieved complete molecular remission with 100% of donor origin hematopoietic cells, but at the cost of induced-GvHD with severe extensive skin involvement (scleroderma and lichenoid lesions, highest modified Rodnan skin score (mRSS): 25), buccal mucosal, and liver involvement. Chronic-GVHD was treated with CNIs (initially cyclosporine and then switched to tacrolimus) plus steroids. No improvement was noticed, so MMF was added, however, only minimal response was achieved. Since ibrutinib, which has been approved by the FDA for the treatment of SR-GvHD [5], and ECP were both not available at that time in our department, as next line of GvHD treatment, we opted to initiate the patient on ruxolitinib (10 mg bid). Following a month of treatment, the drug was discontinued due to GvHD progression (skin-liver). Subsequently, bortezomib 1.3 mg/m^2^ (days 1,8,15,22) every 35 days plus MMF plus steroids were administered but unfortunately after 2 cycles, the patient showed stable GvHD findings, while maintaining steroid dependency. Because of their different mechanism of action, we next decided to combine bortezomib (in the previous administered dose) plus ruxolitinib at the dose of 5 mg bid along with MMF and steroids (dose 1.5 mg/kg daily). After 3 months of treatment with the above combination, marked skin improvement was noticed; lichenoid lesions disappeared, sclerodermatosis was improved (mRSS:12), and liver enzymes became almost normal (Fig. 1a, b, c). Noticeably, the steroids dose was gradually tapered by 75%, while MMF was discontinued. He continued on bortezomib plus ruxolitinib at the aforementioned doses and low-dose steroids (10 mg every other day). During the treatment period, the patient did not experienced any significant Grade ≥ 2 toxicity, including neuropathy or myelotoxicity. The most recent evaluation for his primary disease status confirmed complete molecular remission along with 100% donor chimerism.

**Fig. 1:**
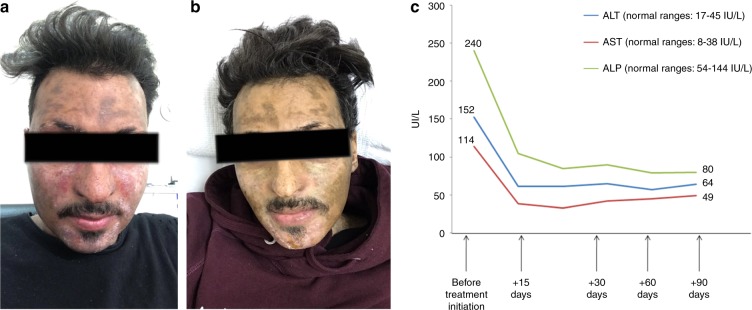
GvHD-related facial skin lesions and functional liver enzymes tests during ruxolitinib-bortezomib combination treatment: a) facial skin lessions before treatment with ruxolitinib–bortezomib combination, b)  facial skin lesions 4 months after treatment with ruxolitinib–bortezomib combination. c) Functional liver tests before and at 1, 2, and 4 months after initiation of ruxolitinib–bortezomib combination

Despite the improvements in its prophylaxis and treatment, SR-cGvHD, continues to pose a major hurdle to ultimately improve outcome of alloSCT [6]. Especially the induced-cGvHD, which our patient experienced, is considered one of the most difficult forms of GvHD to manage [7].

JAK signaling has been proven to be a key regulator of inflammation and tissue damage in GvHD, transducing inflammatory signaling downstream of cytokines and regulating development and function of immune cells including antigen-presenting cells (APC) and T cells. Recently JAK-pathway inhibition was proposed as a potential target for the treatment of SR-GvHD [8]. Zeiser et al. [3] and Khoury et al. [9] reported promising results, with an approximately 85–90% overall response rate and 7% complete remission rate in patients who had previously received more than two lines of treatment for SR-GvHD. However, in our patient the combination of ruxolitinib plus MMP plus steroids proved to be ineffective.

The proteasome-inhibitor bortezomib, through inhibition of nuclear factor kappa-B (NF-kB), results in depletion of proliferating alloreactive T cells, decrease of type-1 helper T-cell cytokine production, and inhibition of APC function, while demonstrating limited influence on the number and function of regulatory T cells, the important mediators for GvHD control [4]. In a limited number of studies bortezomib demonstrated noticeable responses in patients with SR-GvHD [4]. In our patient, additionally to steroids, we initiated bortezomib, according to the regimen previously described [10]. Unfortunately, this combination of treatment also proved to be unsuccessful in controlling GvHD progression.

Interestingly, the combination of ruxolitinib plus bortezomib resulted in almost normalization of liver function tests and a 50% improvement in the skin sclerotic lesions. Moreover, it was feasible to discontinue MMF while steroids dosing was reduced approximately by 75%.

So far, there are no published data regarding the combination of ruxolitinib plus bortezomib as treatment for SR-GvHD. However, the above combination has already been tested in multiple myeloma cell lines and exhibited significant anti-myeloma activity [11].

It is well known that ruxolitinib affects directly the JAK/STAT pathway and indirectly the PI3K/Akt and Ras/Raf/MAPK pathways, while bortezomib inhibits the NF-kB pathway. We assume that the inhibition of these four major signaling pathways by this drug combination finally resulted in the effective activity against this refractory and steroid-depended GvHD.

In this heavily pretreated patient, the combination of ruxolitinib plus bortezomib proved to be highly effective, also demonstrating no toxicity. Undoubtedly, this is a single case and also other immunosuppressive agents additionally to bortezomib and ruxolitinib were used and therefore, it is  inaccurate to draw definite conclusions regarding the real impact of these agents on the treatment response. However, these promising results indicate that the combination of bortezomib plus ruxolitinib could be an alternative approach for patients with SR-cGvHD and merits further investigation in future prospective clinical trials.

## References

[1] Ruutu T, Gratwohl A, de Witte T, Afanasyev B, Apperley J, Bacigalupo A (2014). Prophylaxis and treatment of GVHD: EBMT-ELN working group recommendations for a standardized practice. Bone Marrow Transplant.

[2] Martin PJ, Rizzo JD, Wingard JR, Ballen K, Curtin PT, Cutler C (2012). First- and second-line systemic treatment of acute graft-versus-host disease: recommendations of the American Society of Blood and Marrow Transplantation. Biol Blood Marrow Transplant.

[3] Zeiser R, Burchert A, Lengerke C, Verbeek M, Maas-Bauer K, Metzelder SK (2015). Ruxolitinib in corticosteroid-refractory graft-versus-host disease after allogeneic stem cell transplantation: a multicenter survey. Leukemia.

[4] Al-Homsi AS, Feng Y, Duffner U, Al Malki MM, Goodyke A, Cole K (2016). Bortezomib for the prevention and treatment of graft-versus-host disease after allogeneic hematopoietic stem cell transplantation. Exp Hematol.

[5] Miklos D, Cutler CS, Arora M, Waller EK, Jagasia M, Pusic I (2017). Ibrutinib for chronic graft-versus-host disease after failure of prior therapy. Blood.

[6] Zeiser R, Blazar BR (2017). Pathophysiology of chronic graft-versus-host disease and therapeutic targets. N Engl J Med.

[7] Dazzi F, Fozza C (2007). Disease relapse after haematopoietic stem cell transplantation: risk factors and treatment. Best Pract Res Haematol.

[8] Schroeder MA, Choi J, Staser K, Di Persio JF (2018). The role of janus kinase signaling in graft-versus-host-disease and graft versus leukemia. Biol Blood Marrow Transplant.

[9] Khoury HJ, Langston AA, Kota VK, Wilkinson JA, Pusic I, Jillella A (2018). Ruxolitinib: a steroid sparing agent in chronic graft-versus-host disease. Bone Marrow Transplant.

[10] Herrera AF, Kim HT, Bindra B, Jones KT, Alyea EP, Armand P (2014). A phase II study of bortezomib plus prednisone for initial therapy of chronic graft-versus-host disease. Biol Blood Marrow Transplant.

[11] De Oliveira MB, Fook-Alves VL, Eugenio AIP, Fernando RC, Sanson LFG, de Carvalho MF (2017). Anti-myeloma effects of ruxolitinib combined with bortezomib and lenalidomide: A rationale for JAK/STAT pathway inhibition in myeloma patients. Cancer Lett.

